# Spleen Dynamic Contrast-Enhanced Magnetic Resonance Imaging as a New Method for Staging Liver Fibrosis in a Piglet Model

**DOI:** 10.1371/journal.pone.0083697

**Published:** 2013-12-20

**Authors:** Li Zhou, Tian-wu Chen, Xiao-ming Zhang, Cheng-jun Li, Zhen-feng Yang, Nan-lin Zeng, Li-ying Wang, Ting Li, Dan Wang, Jie Li, Chun-ping Li, Li Li, Xian-yong Xie

**Affiliations:** 1 Sichuan Key Laboratory of Medical Imaging, and Department of Radiology, Affiliated Hospital of North Sichuan Medical College, Nanchong, Sichuan, China; 2 Department of Anatomy, and Morphometric Research Laboratory, North Sichuan Medical College, Nanchong, Sichuan, China; 3 Department of Radiology, Third Affiliated Hospital of Xinxiang Medical College, Xinxiang, Henan, China; 4 Department of Pathology, Affiliated Hospital of North Sichuan Medical College, Nanchong, Sichuan, China; Brandeis University, United States of America

## Abstract

**Objective:**

To explore spleen hemodynamic alteration in liver fibrosis with dynamic contrast-enhanced magnetic resonance imaging (DCE-MRI), and to determine how to stage liver fibrosis with spleen DCE-MRI parameters.

**Materials and Methods:**

Sixteen piglets were prospectively used to model liver fibrosis staged by liver biopsy, and underwent spleen DCE-MRI on 0, 5th, 9th, 16th and 21st weekend after modeling this disease. DCE-MRI parameters including time to peak (TTP), positive enhancement integral (PEI), maximum slope of increase (MSI) and maximum slope of decrease (MSD) of spleen were measured, and statistically analyzed to stage this disease.

**Results:**

Spearman's rank correlation tests showed that TTP tended to increase with increasing stages of liver fibrosis (*r* = 0.647, *P*<0.001), and that PEI tended to decrease from stage 0 to 4 (*r* = −0.709, *P*<0.001). MSD increased slightly from stage 0 to 2 (*P*>0.05), and decreased from stage 2 to 4 (*P*<0.05). MSI increased from stage 0 to 1, and decreased from stage 1 to 4 (all *P*>0.05). Mann-Whitney tests demonstrated that TTP and PEI could classify fibrosis between stage 0 and 1–4, between 0–1 and 2–4, between 0–2 and 3–4, or between 0–3 and 4 (all *P*<0.01). MSD could discriminate between 0–2 and 3–4 (*P* = 0.006), or between 0–3 and 4 (*P* = 0.012). MSI could not differentiate between any two stages. Receiver operating characteristic analysis illustrated that area under receiver operating characteristic curve (AUC) of TTP was larger than of PEI for classifying stage ≥1 and ≥2 (AUC = 0.851 and 0.783, respectively). PEI could best classify stage ≥3 and 4 (AUC = 0.903 and 0.96, respectively).

**Conclusion:**

Spleen DCE-MRI has potential to monitor spleen hemodynamic alteration and classify liver fibrosis stages.

## Introduction

Liver fibrosis, a common feature of chronic liver disease, has been demonstrated to be at high risk of progress to cirrhosis, portal hypertension, or liver failure [1]. Spleen is a companion solid organ associated closely with liver in the portal system, and participates actively in liver fibrosis progression because of portal hypertension [2]. The spleen histological changes are thought to be the first appearance at the stage of liver bridging fibrosis resulted from the hepatic central vein occlusion and portal hypertension [3]. As liver fibrosis progresses, the histological changes including pulp hyperplasia, congestion and even fibrogenesis occur in the spleen, which could alter spleen hemodynamics [4]–[6]. Although histological evaluation is a gold standard for assessing the severity of the disease, it is limited in clinical application because of its invasive nature, high risk of intraperitoneal hemorrhage and difficult-to-sample [7]. A reproducible and reliable noninvasive method is desirable to evaluate spleen hemodynamic changes with liver fibrosis progression.

Recently, dynamic contrast-enhanced computed tomography, ultrasonography, and nuclear medicine have been used to noninvasively detect spleen hemodynamic changes [8]–[10]. Some investigators used dynamic contrast-enhanced computed tomography to study spleen hemodynamics and demonstrated that the blood flow of the spleen decreased in patients with liver cirrhosis and portal hypertension in comparison with healthy participants [8], [11]. However, Zwiebel et al [9] mentioned that spleen perfusion increased in patients with liver cirrhosis using duplex Doppler ultrasonography. Accordingly, the spleen hemodynamics associated with liver fibrosis and cirrhosis remains no conclusive results. As another noninvasive tool, dynamic contrast-enhanced magnetic resonance imaging (DCE-MRI) could be used to detect hemodynamic changes in parenchyma organs due to its advantages of high spatial resolution and tissue resolution, and lack of ionizing radiation over computed tomography, ultrasonography and nuclear medicine [12], [13]. To our knowledge, no reports focus on utilizing spleen DCE-MRI to evaluate spleen hemodynamics for classifying various liver fibrosis stages. Therefore, attempts were made to use DCE-MRI to dynamically evaluate the spleen hemodynamic alteration due to liver fibrosis and to determine which spleen DCE-MRI parameter could best classify liver fibrosis stages.

## Materials and Methods

### Ethics statement

The experimental protocols were approved by the Institutional Committee for Animal Care of North Sichuan Medical College. This study was carried out in strict accordance with the recommendations in the Guide for the Care and Use of Laboratory Animals of the National Institutes of Health. The animals were kept and provided by the laboratory of animals in our institute.

### Animal preparation

Sixteen experimental mature piglets (6 males, 10 females), weighing between 20.0 kg and 24.0 kg, were used in our study. Modeling liver fibrosis was induced with carbon tetrachloride (CCl_4_) because CCl_4_ has been the toxin which was widely used for the experimental study [14], [15]. To induce liver fibrosis, a mixture solution of 40% CCl_4_ dissolved in vegetable oil was injected at 0.25 ml per kilo weight into inferior peritoneal cavity twice weekly for 16 weeks. Because of the peritoneal chemical adhesions resulted from the intraperitoneal injection, the piglets were then fed with the previous mixture solution (0.75 ml/kg body weight) mixed with maize flour once each day for the subsequent 5 weeks. During the course of modeling liver fibrosis, 5% alcohol water was used as the sole drinking water, and the piglets were fed twice daily with maize flour.

On 0, 5th, 9th, 16th and 21st weekend after the beginning of the modeling, all piglets underwent upper abdomen magnetic resonance (MR) examination under general anaesthesia. The modeling process was paused two days before each MR examination, and was continued after each examination till 21st weekend. The general anaesthesia was induced by intramuscular injection of ketamine (15 mg/kg), and maintained by continuous infusion of ketamine (15 mg/kg/h) and diazepam (0.8 mg/kg/h) through one of the ear edge veins. According to the previous study, percutaneous liver biopsy in the right lobe was used as the standard for confirming liver fibrosis and cirrhosis [16]. The piglets underwent the percutaneous biopsy in right lobe of the liver with an 18-gauge ultrasound-guided core shortly after the MR scanning. When the piglets died during the modeling process before 21st weekend, the dead piglets underwent immediate laparotomy, and the entire liver and spleen were taken out. When the piglets were living on 21st weekend, 1/3 animals were randomly sacrificed by air injection into one of the auricular veins and underwent the laparotomy, and the entire liver and spleen were also taken out shortly after the last liver biopsy. The biopsy specimen as well as postoperative specimen of liver and spleen was used to perform the pathologic evaluation of this disease.

### MRI protocols

The spleen MR study was performed with a 1.5 T scanner (Excite; GE Medical Systems, Milwaukee, WI, USA) using a synergy body phased - array eight channel coils. Before the MR examination, the anterior surface of the thorax and abdomen was shaved to keep good contact between the respiratory triggering and the skin. Subsequently, the piglets were fixed in the supine position. The breathing motion of the upper abdomen was restrained with an abdominal bandage during MR scanning.

Under general anaesthesia, the two-dimensional fast imaging employing steady - state acquisitions were obtained encompassing the spleen as far as possible after routine axial T1- and T2-weighted images were obtained, which were performed for localizing spleen and planning the subsequent DCE-MRI scans. The spleen DCE-MRI with a modified multiphase fast gradient echo train pulse acquisitions were obtained through the upper abdomen encompassing the spleen using the following scanning parameters: repetition time, 14.7 ms; echo time, 3.2 ms; field of view, 34×34 cm; matrix, 256×160; bandwidth, 62.5 kHz; number of signals excitations, 0.5; flip angle, 25°; number of sections, 12; section thickness, 8 mm; slice gap, 1 mm; and acquisition time, 108 s. The DCE-MRI acquisitions started with a bolus intravenous injection of Gadolinium diethylenetriamine-pentaacid (Gd-DTPA Magnevist; Schering, Berlin, Germany) at the rate of 3 mL/s according to 0.15 mmol/kg of body weight with an MR compatible power injector (Spectris MR Injector System; Medrad, Pittsburgh, PA, USA) into the ear edge vein followed by a saline solution flush at the same rate totaling the amount same as the contrast agent. In total, 480 images were continuously obtained within 108 s at rate of approximately 2.7 s for each image using a fast gradient-echo sequence.

### DCE-MRI data analysis

**Figure 1:**
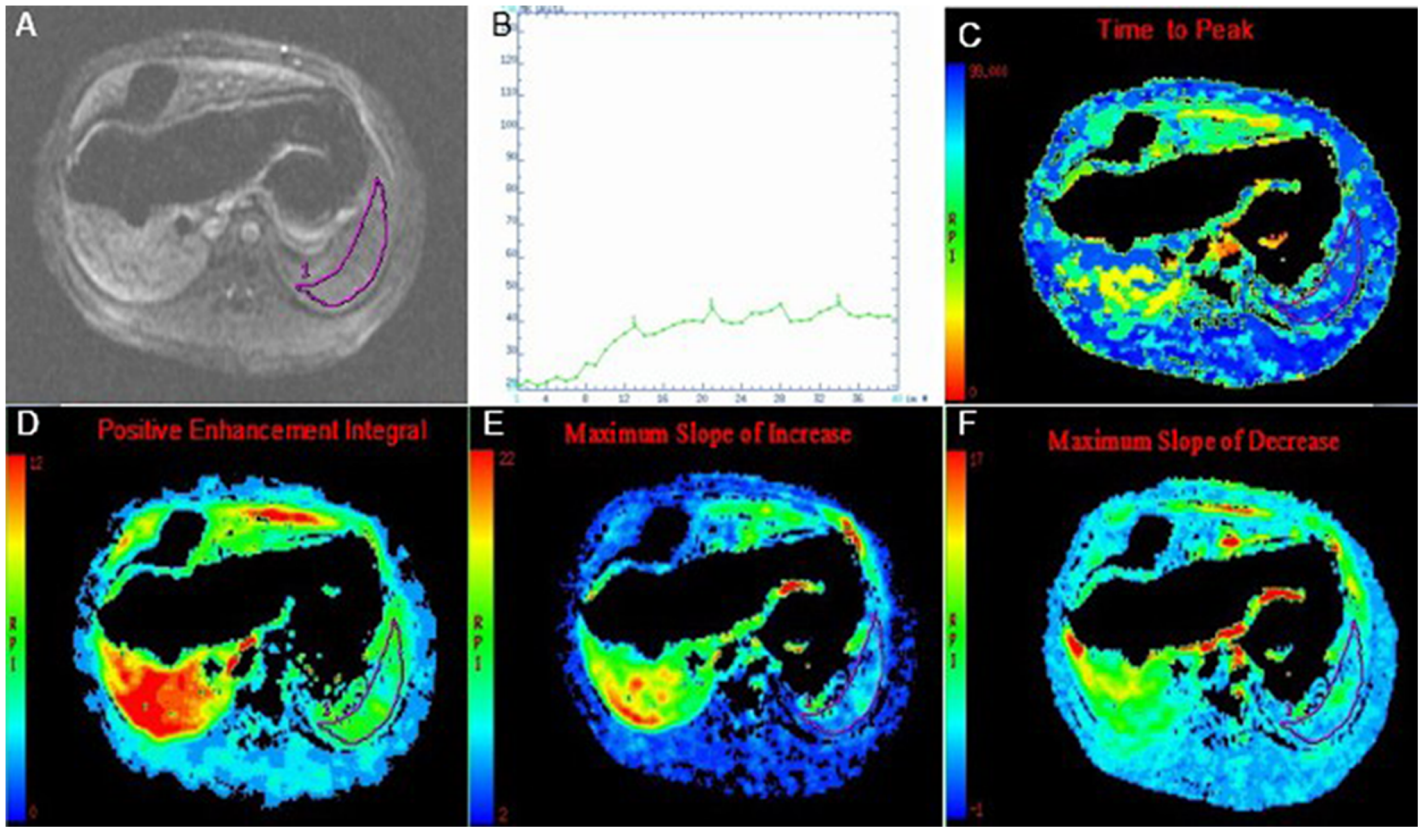
An example of spleen DCE-MRI data measurement. The image (A) shows that region of interest (ROI) has been carefully drawn on dynamic contrast-enhanced magnetic resonance image (DCE-MRI), and the obtained signal-time intensity curves (STC, B) of spleen parenchyma are derived from the standard software package. Graphs show the color maps of time to peak (C), positive enhancement integral (D), and maximum slope of increase (E) and decrease (F).

The DCE-MRI data were transferred to the workstation (GE Advanced Workstation version 4.4-09, Sun Microsystems, Palo Alto, CA, USA) and analyzed with an MR standard software package independently by two radiologists (the corresponding author and the first author with 15 and 3 years of experience in abdominal MR imaging, respectively). The acquired imaging data were of satisfactory quality for the analysis. The two radiologists measured the DCE-MRI parameters by manually placing regions of interest (ROIs) on consecutive three maximum sections of spleen. The mean value of each DCE-MRI parameter was calculated from the 3 different measurements. Motion correction and noise reduction were performed for all data using possession threshold. The ROIs were drawn manually as large as possible within the spleen to allow for regional heterogeneity of perfusion but not so large as to approach the margin of the spleen to avoid the creation of partial volume effects. Care was taken to exclude spleen hilar blood vessels when drawing the ROIs within the spleen (Figure 1A). Signal intensity time curve (STC, Figure 1B) was derived, and spleen DCE-MRI parameters including time to peak (TTP, Figure 1C), positive enhancement integral (PEI, Figure 1D), maximum slope of increase (MSI, Figure 1E), and maximum slope of decrease (MSD, Figure 1F) were automatically generated by the commercial software based on the curve analysis model [17], [18]. According to this model, TTP was defined as the time from the time point when enhancement could be detectable to the time when the maximum signal intensity (SImax) appeared in the spleen. PEI represented the spleen blood perfusion in the ROIs, which was obtained by calculating the area under STC. MSI (or MSD) could be computed by the formula: MSI or MSD  = (SI_2_ − SI_1_)/t, where SI_2_ and SI_1_ represented signal intensity at two different time points in increase (or decrease) STC with relative maximum ratio, respectively, and t represented time interval between the two time points. To reduce information bias, the two observers were instructed with a separate teaching dataset on how to follow the same rules in the data analysis. To test the inter- and intra-observer reproducibility of measurements of spleen DCE-MRI parameters, these parameters were estimated repeatedly by the previous radiologists, and 3 weeks of a delay was established between the two measurement sessions.

### Histopathology evaluation

Liver specimens obtained by biopsy and laparotomy together with spleen specimen obtained by laparotomy were fixed in formalin, embedded in paraffin, sectioned, and examined with light microscopy after standard hematoxylin - eosin (H&E). The hepatic tissue specimen was also stained with Masson-trichrome staining for staging liver fibrosis. Two experienced pathologists (the 12th and 13th author with 11 and 37 years of experience in hepatopathology, respectively) scored the pathologic specimens working in consensus, who had no prior clinical and radiological information of the animals. Because the appropriate liver morphology and histology of experimental piglets were similar to that of human, liver fibrosis stages in this study were based on the METAVIR classification for human as follows: stage 0, no fibrosis; stage 1, portal fibrosis; stage 2, periportal fibrosis; stage 3, septal fibrosis; and stage 4, cirrhosis [19].

### Statistical analysis

SPSS statistical package version 17.0 was used for the statistical computations. Descriptive statistics included means and standard deviation of each DCE-MRI parameter. A *P* value of less than 0.05 was considered to represent a significant difference.

The correlation of each DCE-MRI parameter with liver fibrosis stages was performed by using the Spearman's rank correlation coefficient. Because the DCE-MRI parameters were of skewed distribution, nonparametric Mann-Whitney tests were used to compare these parameters between fibrosis stages; and when *P* value was less than 0.05, the *P* value corresponding to the DCE-MRI parameter was further corrected for multiple testing by Bonferroni method. The cutoff values of the spleen DCE-MRI parameters were then analyzed with receiver operating characteristic (ROC) analysis for classification of liver fibrosis stage ≥1, ≥2, ≥3 and 4. The diagnostic performance of the spleen parameters in classifying liver fibrosis stages was assessed with the area under the ROC curves (AUC).

Additionally, inter- and intra-observer variability for the spleen DCE-MRI parameters of liver fibrosis stage 3 were randomly chosen to statistically assess the general variability for all stages by intra-class correlation coefficient (ICC) mode and Pearson correlation coefficient, respectively [20]. To test the histopathology consistency of liver fibrosis stages by the last percutaneous biopsy and by the laparotomy for the dead animals before the 21st weekend and randomly killed animals on the 21st weekend, the agreement for the percutaneous biopsy and the laparotomy was expressed with *k*-value statistics. These statistics was generally interpreted as follows: *k*<0.41, poor agreement; *k* = 0.41–0.60, moderate; *k* = 0.61–0.80, good; and *k* = 0.81–1.00, excellent [21].

## Results

### Animal models and histological findings

**Figure 2:**
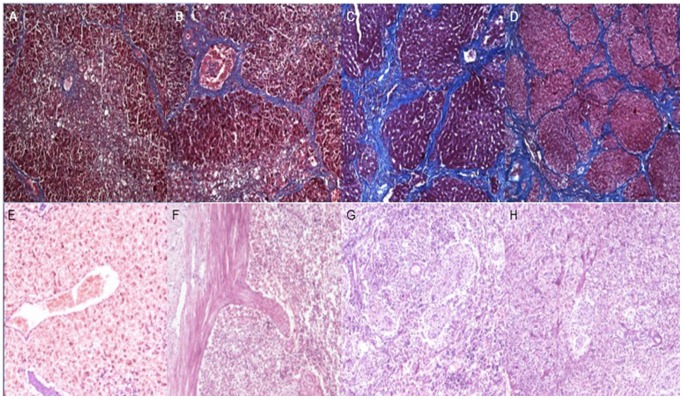
Histology of liver fibrosis stages and the corresponding spleen histological findings. Liver microscopic images (Masson-trichrome, original magnification ×100) show liver fibrosis stage 1 (A), 2 (B), 3 (C) and 4 (D). Spleen microscopic images (hematoxylin-eosin, ×100) show the spleen pulp congestion, hyperplasia and fibrosis infiltration corresponding to the liver fibrosis stages 1 (E), 2 (F), 3 (G) and 4 (H).

In this cohort, 1 piglet died between week 5 and 9, 2 piglets died between week 9 and 16, and 4 piglets died between week 16 and 21 during the follow-up period. Among the dead animals, 2 piglets died of causes unrelated to the experimental protocol, and 5 died of toxic reaction. In the dead animals, 4 piglets had been successfully induced to liver cirrhosis, and 3 had liver fibrosis. In the surviving piglets, different liver fibrosis stages were confirmed on the follow-up weekends according to the METAVIR classification system, and we considered the weekend on which liver fibrosis was initially confirmed by pathology as the time when the fibrosis occurred during the follow-up (Table 1). With the progression of liver fibrosis, this fibrosis was staged as 1 (Figure 2A), 2 (Figure 2B), 3 (Figure 2C) and 4 (Figure 2D), and the piglets according to fibrosis stages are given in Table 1. And there was a good agreement between fibrosis stages determined by the last percutaneous biopsy and by the laparotomy for the dead animals before the 21st weekend and randomly killed animals on the 21st weekend (*k* = 0.80; 95% confidence interval (CI), 0.75–0.84). Spleen pulp congestion, hyperplasia and fibrosis infiltration were found in the dead or sacrificed piglets according to the fibrosis stages (Figure 2E, F, G and H). In addition, neither seroperitoneum nor collateral circulation was found in all animals when laparotomy was performed.

**Table 1:** Body weight and liver fibrosis stage in surviving piglets on follow-up weekends after the modeling of this disease.

Weekend (*n*)	Body weight (kg)	S0	S1	S2	S3	S4
0 (16)	22.0±1.31	16	0	0	0	0
5 (16)	24.1±2.41	3*	8	5	0	0
9 (15)	24.0±2.45	0	3	7	5	0
16 (13)	25.3±2.81	0	1*	1	7	4
21 (9)	23.5±1.78	0	1*	1*	1	6

Notes: *Animals with liver fibrosis at the current stage are same as those at the immediately previous stage during the follow-up period. S0, S1, S2, S3 and S4 represent liver fibrosis stage 0, 1, 2, 3 and 4, respectively.

On 0, 5th, 9th, 16th and 21st weekend, mean body weight of the surving animals is shown in Table 1. There was no significant difference between any two weekends in terms of body weight in each piglet.

### Inter- and intra-observer agreements of DCE-MRI parameter measurements

Based on the spleen DCE-MRI parameters of liver fibrosis stage 3 obtained independently and repeatedly by the two radiologists, the inter-observer measurements of these parameters are TTP (ICC, 0.90; 95%CI, 0.86–0.96), PEI (ICC, 0.91; 95%CI, 0.88–0.95), MSI (ICC, 0.90; 95%CI, 0.86–0.95) and MSD (ICC, 0.89; 95%CI, 0.84–0.94). The Pearson correlation coefficient showed good agreements between the measurements of the intra-observers for TTP (*r* = 0.91, *P*<0.001), PEI (*r* = 0.95, *P*<0.001), MSI (*r* = 0.90, *P*<0.001) and MSD (*r* = 0.89, *P*<0.001). Therefore, inter- and intra-observer variability of the parameters was small, and the mean value of two measurements across the two observers was used as the final values.

### Spleen DCE-MRI parameters corresponding to liver fibrosis stage

**Figure 3:**
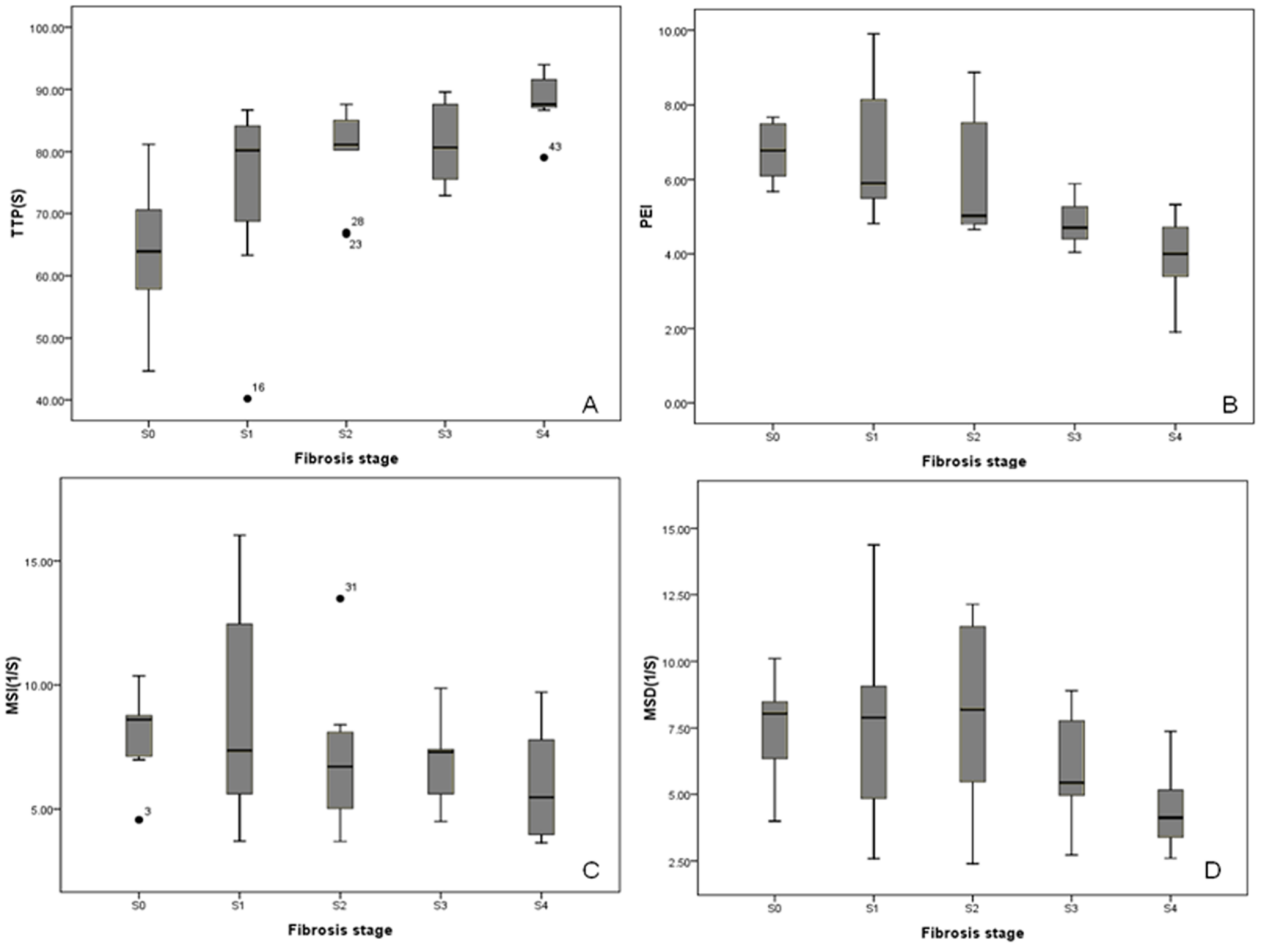
Distributions of DCE-MRI parameters at various fibrosis stages. Box plots show distributions of time to peak (TTP, A), positive enhancement integral (PEI, B), maximum slope of increase (MSI, B), and maximum slope of decrease (MSD, D) corresponding to liver fibrosis stages.

Spleen DCE-MRI parameters corresponding to stage of liver fibrosis are shown in Figure 3. TTP were observed to increase from liver fibrosis stages 0 to 4 (*r* = 0.647, *P*<0.001). PEI decreased from stage 0 to 4 (*r* = −0.709, *P*<0.001). MSD increased from stage 0 to 2, and then decreased from stage 2 to 4 (*r* = −0.345, *P* = 0.018). MSI increased slightly from stage 0 to 1, and decreased from stage 1 to 4 (*P* = 0.056). As shown in Table 2, TTP could discriminate liver fibrosis stages between stage 0 and 1, 2, 3 or 4, and between stage 4 and 1, 2 or 3 (all *P*<0.05). PEI was found to be different between liver fibrosis stage 0 and 3 or 4, between stage 1 and 3 or 4, and between stage 2 and 4 (all *P*<0.001). MSD could discriminate 0, 1 or 2 from 4 (all *P*<0.001). However, MSI could not classify any two liver fibrosis stages with statistical significance.

**Table 2:** Dynamic contrast-enhanced magnetic resonance imaging parameters of spleen corresponding to the stage of liver fibrosis.

	S0 (*n* = 16)	S1 (*n* = 11)	S2 (*n* = 13)	S3 (*n* = 13)	S4 (*n* = 10)
TTP (s)	63.97 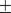 12.08	74.4 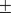 13.19^a^	79.67 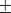 7.76^a^	81.08 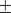 6.11^a*^	88.29 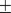 4.9^a*b*cd^
PEI	6.78 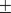 0.76	6.31 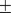 2.39	6.09 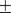 1.74	4.94 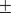 0.58^a*b*^	3.78 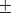 0.46^a*b*c^
MSI (1/s)	8.01 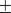 1.6	9.04 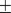 4.29	7.0 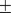 2.9	6.93 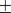 1.7	6.05 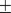 2.38
MSD (1/s)	7.36 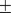 1.8	7.58 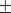 3.58	8.12 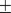 3.41	5.81 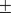 2.17	4.45 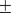 1.63^abc^

Notes: TTP  =  time to peak, PEI  =  positive enhancement integral, MSI  =  maximum slope of increase, and MSD  =  maximum slope of decrease. S0, S1, S2, S3 and S4 represent liver fibrosis stage 0, 1, 2, 3 and 4, respectively; a, b, c and d represent different from S0, from S1, from S2, and from S3, respectively (all *P*<0.05). ^*^ denotes the significance after Bonferroni correction.

The Mann-Whitney tests (Table 3) showed that there were significant differences in TTP and PEI between grouped stages 0 and 1–4, between stages 0–1 and 2–4, between stages 0–2 and 3–4, or between stages 0–3 and 4. MSD could classify between stages 0–2 and 3–4, or between stages 0–3 and 4 (all *P*<0.05). MSI could not classify any two liver fibrosis grouped stages.

**Table 3:** Utility of dynamic contrast-enhanced magnetic resonance imaging parameters of spleen to discriminate between grouped stages of liver fibrosis.

	S0 vs. S1–4	S0–1 vs. S2–4	S0–2 vs. S3–4	S0–3 vs. S4
TTP (s)	0.001*	<0.001*	0.001*	0.001*
PEI	0.003*	<0.001*	<0.001*	0.001*
MSI (1/s)	0.238	0.054	0.164	0.16
MSD (1/s)	0.297	0.138	0.006*	0.011*

Notes: TTP  =  time to peak, PEI  =  positive enhancement integral, MSI  =  maximum slope of increase, and MSD  =  maximum slope of decrease. S0, S0–1, S0–2, S0–3, S1–4, S2–4, S3–4 and S4 represent liver fibrosis stage 0, 0–1, 0–2, 0–3, 1–4, 2–4, 3–4 and 4, respectively. * denotes significant in the parameters between the grouped stages (*P* < 0.05), and all the comparisons denote significance after Bonferroni correction.

### ROC analysis for classifying liver fibrosis stage

For staging liver fibrosis, the cutoff values of spleen DCE-MRI parameters as well as the AUC, sensitivity and specificity are shown in Table 4. TTP and PEI could differentiate liver fibrosis stage ≥1, ≥2, ≥3 and 4. MSD could classify liver fibrosis stage ≥3 and 4. Moreover, spleen TTP had larger AUC for classifying liver fibrosis stage ≥1 and ≥2 compared with spleen PEI. The best parameter that could be used to classify liver fibrosis stage ≥3 and 4 was PEI among all the spleen DEC-MRI parameters.

**Table 4:** Receiver operating characteristic analysis of spleen dynamic contrast-enhanced magnetic resonance imaging parameters for detection of fibrosis stages ≥1, ≥2, ≥3 and 4.

Cutoff value	Fibrosis stages	AUC	Sensitivity (%)	Specificity (%)
TTP (s)				
70.82	≥1	0.851	80.6	81.8
73.75	≥2	0.783	80	63.6
80.46	≥3	0.8	68.8	61.3
86.36	4	0.886	85.7	85
PEI				
5.95	≥1	0.778	90.9	75
5.89	≥2	0.776	72.7	84
5.46	≥3	0.903	77.4	99.4
4.75	4	0.968	95	85.7
MSD (1/s)				
5.45	≥3	0.745	74.2	62.5
4.75	4	0.8	85	71.4

Notes: TTP  =  time to peak, PEI  =  positive enhancement integral, MSI  =  maximum slope of increase, and MSD  =  maximum slope of decrease. AUC  =  area under the receiver operating curve.

## Discussion

Liver fibrosis is now demonstrated to be a dynamic process with potential for regression at least clinically and possibly morphologically due to effective and appropriate antifibrotic therapy or eliminating the causes [22]. Patients with liver fibrosis stages 2–4 could obtain well-preserved liver function and restoration of normal architecture by the clinical interventions whereas patients with liver fibrosis stage ≤1 should not receive the interventions but be monitored to prevent liver fibrosis progression. Staging liver fibrosis is critically important for the above-mentioned treatments decision making [22]–[24]. Prior to the onset of advanced liver fibrosis and cirrhosis, portal hypertension occurs, which will initially and directly induce spleen dynamic circulatory alterations [3]–[6], [8], [9]. We could presume that the evaluation of spleen dynamic circulatory alterations might be especially important for staging liver fibrosis. In this study, we used spleen DCE-MRI to investigate how to stage liver fibrosis. Our study showed that spleen TTP had a positive correlation, and PEI and MSD had negative correlations with liver fibrosis stages while MSI tended to decrease without significant changes. Among all the parameters, TTP had best performance to classify liver fibrosis stage ≥1 and ≥2, and PEI was best to classify fibrosis stage ≥3 and 4.

As shown in this study, the correlations of spleen DCE-MRI parameters with liver fibrosis stages could be explained by the spleen hemodynamic changes. With liver fibrosis progression, hepatic central vein occlusion and increasing portal pressure result in spleen congestion, and the network of sinuses and cords induces the production of collagen in the basement membrane of the spleen vessels, finally leading to direct fibrosis infiltration of the spleen [3], [4], [25]. This congestion and tissue hyperplasia condition would decrease the blood volume and prolong the transit time of blood through the spleen [8]. This progress could result in spleen TTP prolonging and PEI decreasing as liver fibrosis progresses. This congestion and tissue hyperplasia condition also reduce the blood flow of spleen, leading to the decrease of MSI or MSD with the progression of liver fibrosis. Another fact to explain the findings might be the unique natures of spleen complex microcirculation: open and closed circulation within spleen [26]. The percentage of spleen blood flow entering the open circulation may increase in liver fibrosis. Blood cells filter through the pulp cords in the open circulation, and then reenter the venous sinuses crossing the windowed sinusoids, which also results in the transit time of blood cells prolonging and the spleen circulation slowing down [4].

In addition, we demonstrated that MSI, reflecting spleen artery flow rate, tended to decrease with the progress of liver fibrosis without statistical significance. However, Motosugi et al [8] performed multi-organ computed tomography perfusion study in the abdomen and reported that the blood supply from the spleen artery decreased in cirrhotic patients with statistical significance in comparison with healthy participants. Our findings are inconsistent with this published article, and the possible explanation might be because the chronic liver disease was severe cirrhosis in the enrolled patients of this published paper while this disease in our study was liver fibrosis at different stages.

Because of the correlations of spleen TTP, PEI and MSD with liver fibrosis stages with statistical significance, we utilized the Mann-Whitney tests to further determine whether the DCE-MRI parameters could discriminate liver fibrosis stages. Our study showed that each of these parameters could distinguish specific or grouped stages of this disease. Subsequently, the ROC analysis also showed that spleen TTP, PEI and MSD could be used to classify liver fibrosis stages. Spleen TTP had larger AUC in comparison with PEI for classifying liver fibrosis stage ≥1 and ≥2, and PEI had better performance in identifying stage ≥3 and 4 compared with TTP or MSD. Our results could be explained by the spleen main pathologic changes at different stages of liver fibrosis: the passive congestion results from portal pressure increasing at the early stage of liver fibrosis, which mainly evokes spleen TTP prolonging at stages ≥1 and ≥2; and spleen hyperplasia and fibrosis infiltrations at the advanced stages of liver fibrosis and early cirrhosis lead to the decrease of spleen PEI at stages ≥3 and 4 [27]. Therefore, TTP could be recommended for classifying liver fibrosis stages ≥1 and ≥2, and PEI could be recommended for discriminating stage ≥3 and 4.

Our study has several limitations. Firstly, our sample size was relatively small. Therefore, a larger number of samples will be involved in our further study to confirm spleen DCE-MRI parameter for classifying stages of liver fibrosis. Secondly, our study was a kind of indirect examination on spleen to evaluate and compare the spleen hemodynamic with the liver tissue itself, but our findings provided some useful information that spleen DCE-MRI parameters could classify the stages of fibrosis. Thirdly, our study was based on an animal experiment, but our findings suggested that the study might be a good approximation for clinical application.

In conclusion, spleen DCE-MRI is a newly noninvasive method for evaluating spleen hemodynamic alteration at different liver fibrosis stages. Spleen TTP can be recommended as a predictive marker for estimating fibrosis stage ≥1 and ≥2, and PEI can be recommended to classify fibrosis stage ≥3 and 4. We hope that our findings will be helpful for a similar study in clinical settings.
